# Use, tolerability and compliance of spironolactone in the treatment of heart failure

**DOI:** 10.1186/1472-6904-11-4

**Published:** 2011-05-20

**Authors:** Jean Lachaine, Catherine Beauchemin, Elodie Ramos

**Affiliations:** 1 Faculty of Pharmacy, University of Montreal, Montreal, Quebec, Canada; 2 Health Economics and Outcomes Research, Pfizer Canada Inc., Kirkland, Quebec, Canada

## Abstract

**Background:**

Risk of morbidity and mortality in patients with severe heart failure (HF) is reduced by blockade of aldosterone receptors with spironolactone. However, benefits of spironolactone are potentially limited by treatment compliance and adverse events profile. The aim of this study was to estimate use of spironolactone by patients with HF, incidence of key adverse events, and patient compliance.

**Methods:**

This study was performed using data from the Quebec provincial medical and drug plans (*Régie de l'Assurance Maladie du Québec*, RAMQ) for patients who had a diagnosis of HF. Relative incidence of gynecomastia and hyperkalemia was estimated for users and non-users of spironolactone. Treatment adherence was estimated for users of spironolactone and compared to adherence with angiotensin converting enzyme (ACE) inhibitors, beta-blockers (β-blockers), and angiotensin receptor blockers (ARBs).

**Results:**

RAMQ data were obtained for a total of 82,018 patients with a diagnosis of HF. Of these patients, 59.9% used an ACE inhibitor, 59.5% used a beta-blocker, 28.4% used an ARB, and 15.1% (n = 12,344) used spironolactone. Despite underestimation due to limitation of the database, the documented incidence of hyperkalemia (3.3% versus 1.4%) and gynecomastia (1.8% versus 0.7%) was significantly higher in spironolactone users than non-users (p < 0.001). Treatment compliance was significantly lower with spironolactone compared to ACE inhibitors, β-blockers, and ARBs (45.6% versus 56.1%, 59.7%, and 57.0%, respectively; p < 0.001). Persistence to treatment over a one-year period was also lower with spironolactone compared to ACE inhibitors, β-blockers, and ARBs (50.7% versus 64.5%, 70.4%, and 66.3%, respectively; p < 0.001).

**Conclusion:**

Use of spironolactone is associated with an incidence of adverse events, which may have an impact on treatment compliance.

## Background

Heart failure (HF) is a chronic condition defined as the inability of the heart to maintain a sufficient pumping activity to meet the body's metabolic demands under normal filling pressure [1]. It is usually characterized by breathlessness (including orthopnea and dyspnea), effort intolerance, swelling of the legs, and fluid retention [2]. In Canada, HF affects over 500,000 individuals, with approximately 50,000 incident cases diagnosed yearly [3]. A retrospective Canadian study comprising 72 patients who had two or more visits to a heart failure clinic revealed that 71% and 21% of subjects were having a severe HF (NYHA class III and IV respectively, with a mean ejection fraction of 31%) [4]. HF is associated with a substantial clinical and economical burden. In Canada, more than 54,330 patients were hospitalized for HF between 2005 and 2006. Moreover, 4,430 Canadians with HF died in 2004, representing 2% of all deaths [5]. The average cost incurred by HF patients during their last six months of life was $27,983 in Canadian dollars in 2006 [6].

Aldosterone, a steroid hormone known to enhance sodium retention and potassium secretion, plays a major role in the pathophysiology of heart failure (HF). According to the results of the Randomized Aldactone Evaluation Study (RALES), the use of spironolactone, a competitive aldosterone antagonist, an angiotensin converting enzyme (ACE) inhibitor, a loop diuretic, and in most cases digoxin, significantly reduces the risk of mortality and morbidity in patients with severe HF (NYHA class III-IV, LVEF ≤35%) [7]. Results of this randomized trial suggested that adding spironolactone to standard treatment for severe HF reduces death rates (all causes and cardiac causes) and hospitalization rates due to cardiac conditions. In fact, plasma aldosterone concentrations in HF patients may reach 20 times the normal level, because of both increased aldosterone production and decreased rate of hepatic clearance [8]. Thus, ACE inhibitors are not sufficient to suppress the production of aldosterone in HF patients, hence the importance of adding an aldosterone antagonist, such as spironolactone, to severe HF therapy [9].

However, spironolactone is associated with serious adverse events, including hyperkalemia and gynecomastia [10,11]. Consequently, clinical benefits related to the use of spironolactone are potentially limited by the adverse events profile, which may lead to premature treatment cessation and poor treatment adherence.

The purpose of this study was to estimate, in real clinical practice, the utilization rate of spironolactone in patients with HF, the incidence of key adverse events (hyperkalemia and gynecomastia), and patient adherence to spironolactone in terms of compliance and persistence.

## Methods

This study was performed using data from the database of the Quebec provincial health plans (*Régie de l'Assurance Maladie du Québec*, RAMQ). Like other Canadian provinces, Quebec has a universal health care program that covers physician services and hospitalization for the entire population. This universal health program is complemented, for a large proportion of the population, by a public drug plan. The provincial drug reimbursement program covers all people aged 65 and over, beneficiaries of the social assistance program, and individuals who do not have access to a private medication insurance plan. As opposed to the health care plan, for which all costs are covered, the drug plan involves limited financial participation on the part of the beneficiaries. More than 40% of Quebec's population is covered by the drug program [12].

The RAMQ medical services database contains information from physicians' claims for services provided within and outside the hospital. The RAMQ pharmaceutical services database includes information from pharmacists' claims for dispensed medications reimbursed by the program, but not for medications received in a hospital. In addition, data obtained from the RAMQ include an encrypted patient identifier, which enables linkage of individual patient information while preserving anonymity. Because patient data used in this study were anonymous, ethical approval was not required for data analyses.

Data on medical and pharmaceutical services from January 2000 to September 2008 were obtained from the RAMQ for patients who had a diagnosis of HF (*The International Classification of Diseases, Ninth Revision*: ICD-9 codes: 4280 - 4289) [13] during that period. Patient characteristics were described in terms of gender, age, and comorbidities. Comorbidity level was estimated by calculating a chronic disease score, based on the Von Korff score, and updated to account for the medications introduced since its initial development [14]. Occurrences of selected medications use during the study period were used to calculate the chronic disease score. Use of spironolactone, ACE inhibitors, beta-blockers (β-blockers), and angiotensin receptor blockers (ARBs) was also estimated. In addition, use of spironolactone with an ACE inhibitor, an ARB, or a β-blocker was defined as concomitant when the usage overlap period for the two medications was at least 60 days.

Relative incidence of adverse events associated with the use of spironolactone, such as gynecomastia or mastopathy (ICD-9 codes: 6110 - 6119) and hyperkalemia (ICD-9 code: 2767) [13], was estimated by the occurrence of the corresponding ICD-9 codes found in the medical services data for users and non-users of spironolactone.

Treatment adherence was estimated for initial users of standard therapy (ACE inhibitors, ARBs, or β-blockers) or spironolactone. Patients were considered new users if the first script for one of these treatments had not been preceded by any other script for the same treatment in the 12 previous months. In this analysis, treatment adherence comprises two specific dimensions: compliance to treatment and persistence to treatment. Compliance refers to the consistency and accuracy with which the patient follows the recommended treatment regimen, while persistence represents the long-term continuation of treatment.

For each study subject, treatment compliance was estimated by calculating the ratio of effective treatment duration over expected treatment duration. Effective treatment duration was estimated by the quantity of medication received and the corresponding number of days of treatment. Treatment compliance was estimated over a one-year period. Patients were considered compliant if their compliance ratio was equal to or greater than 80%. In a complementary analysis, patients were considered compliant if their compliance ratio was equal to or greater than 50%.

Treatment persistence was estimated over a 3-, 6-, and 12-month period. Patients were considered persistent for as long as the treatment (spironolactone, ACE inhibitors, ARBs, or β-blockers) had not definitively ceased. Treatment cessation was determined when the patients had not received treatment for at least six months.

Differences in incidence of adverse events in spironolactone users and non users were tested for significance using Pearson's chi square test. This statistical test was also performed to compare treatment compliance and persistence with spironolactone and standard therapy (ACE inhibitors, ARBs, β-blockers).

## Results

During the period from January 1, 2000 to September 30, 2008, a total of 238,721 patients had received at least one diagnosis of HF. In accordance with the RAMQ's restrictions on the number of subjects available for analysis by external parties, data were obtained on a random sample of 82,018 of these patients. Of this sample, 15.1% (n = 12,344) used spironolactone. There was a higher proportion of men among spironolactone users, as opposed to the overall HF group, where the proportion of women was higher. In addition, comorbidity level, estimated by the modified Von Korff score, was higher for patients using spironolactone. A large proportion of spironolactone users received concomitant treatment with ACE inhibitors and β-blockers, and to a lesser extent with ARBs (Table 1).

**Table 1:** Patient characteristics

	CHF n = 82 018	CHF using spironolactone n = 12,344
Age group		
<20	518 (0.6%)	11 (0.1%)
20-39	1,366 (1.7%)	62 (0.5%)
40-59	6,114 (7.5%)	716 (5.8%)
60-79	31,499 (38.4%)	5,093 (41.3%)
80+	42,521 (51.8%)	6,462 (52.3%)
Average age (SD)	77.2 (14.1)	78.5 (11.4)

Gender		
- male	39,132 (47.7%)	6,368 (51.6%)
- Female	42,886 (52.3%)	5,976 (48.4%)

Average Von Korff score (SD)	7.6 (3.4)	8.8 (2.9)

Selected drug use		
- ACE	49,140 (59.9%)	9,493 (76.9%)
- ARB	23,326 (28.4%)	4,455 (36.1%)
- β-blocker	48,772 (59.5%)	9,260 (75.0%)

Concomitant use with		
- ACE		6,702 (54.3%)
- ARB		2,654 (21.5%)
- β-blocker		7,217 (58.5%)

Incidence of hyperkalemia, breast complications in men, and more specifically gynecomastia, was significantly higher in spironolactone users than in non-users (Table 2).

**Table 2:** Incidence of adverse events

	Spironolactone users	Spironolactone non-users
Hyperkalemia	408/12,344 (3.3%)	955/69,674 (1.4%)*

Breast complications in men	158/6,368 (2.5%)	380/32,764 (1.2%)*

Breast hypertrophy in men (gynecomastia)	117/6,368 (1.8%)	233/32,764 (0.7%)*

* p < 0.001 (Pearson's chi square test)

Over a one-year period, treatment compliance was significantly lower with spironolactone compared to ACE inhibitors, β-blockers, and ARBs, according to both 80% and 50% thresholds (Table 3). Moreover, persistence to treatment after 3-, 6-, and 12-month periods was also lower with spironolactone compared to ACE inhibitors, β-blockers, and ARBs (Table 4).

**Table 3:** Treatment compliance

	Compliance	
	
	50%	80%
ACE	66.4%	56.1%

ARB	68.1%	57.0%

β-blockers	71.1%	59.7%

Spironolactone	58.4%*	45.6%*

* Spironolactone versus ACE, ARB and β-blockers: p < 0.001 (Pearson's chi square test)

**Table 4:** Treatment persistence

	Persistence		
	
	3 months	6 months	12 months
ACE	83.6%	75.3%	64.5%

ARB	84.8%	77.5%	66.3%

β-blockers	86.3%	80.0%	70.4%

Spironolactone	76.3%*	65.3%*	50.7%*

* Spironolactone versus ACE, ARB and β-blockers: p < 0.001 (Pearson's chi square test)

Diagnosis of HF was received after a diagnosis of acute myocardial infarction (AMI) for 9,288 (11.3%) of the HF patients. Of these HF post-AMI patients, 1,016 (10.9%) used spironolactone after the diagnosis was received, while only 686 (7.4%) patients used spironolactone in the first year following the diagnosis. Of the HF post-AMI patients, 45.4% were compliant over a one-year period, based on the 80% threshold, and 47.6% showed persistence to treatment after a 12-month period.

## Discussion

Although spironolactone has proven beneficial for patients with HF, only a small proportion of HF patients in this study (15.1%) used this medication. As previously reported [7], this study found that spironolactone use was associated with higher incidence of adverse events, including hyperkalemia and gynecomastia. Treatment adherence was less than optimal for all medications considered in this analysis, particularly for spironolactone. In fact, both compliance and persistence with spironolactone were significantly lower compared to standard therapies (ACE inhibitors, β-blockers, or ARBs). Similarly, treatment persistence related to spironolactone was found to be a major issue in the study by Bouvy and al., where 40.8% and 58.8% of patients discontinued treatment after 6 months and 7 years, respectively [10].

As this study used data from an administrative database, it enabled the inclusion of a very large number of subjects who are assumed to accurately represent a real-world clinical setting, contrary to a clinical trial with a small sample size and controlled clinical environment. However, as in other studies based on administrative databases, there are some inherent limitations. It is assumed that the reimbursed medications retrieved from the databases were taken by the patient, although this may not always be the case. This could result in overestimation of treatment adherence. Moreover, medications received while hospitalized and medications that were not reimbursed by the drug plan were not taken into account.

Another limitation of this study is related to the use of ICD-9 codes to identify patients with HF as well as occurrence of hyperkalemia and breast complications. In Quebec, physicians are not required to record an ICD-9 code when meeting their patients, although the vast majority of them do. In addition, because only a single ICD-9 code can be recorded on the form for each claim submitted to the RAMQ for reimbursement of a medical service, the prevalence of a specific diagnosis can be underestimated when patients present more than one medical condition. For example, the diagnosis of gynecomastia in HF patients may not be captured when a diagnosis of HF was first reported. In fact, the incidence of gynecomastia in spironolactone users in the present study (1.8%) was much lower than that reported in the RALES study (9.0%) [7]. As opposed to data from clinical studies, the severity of adverse events is unknown in studies based on claims data. For example, cases of serious hyperkalemia (>6 mmol/L) were reported in the RALES study, whereas only the overall incidence of hyperkalemia could be estimated in the present study [7]. Moreover, studies conducted by the RALES investigators found that hyperkalemia was a dose-dependent effect of spironolactone, as well as gynecomastia, and required monitoring of the patient [7,15]. This monitoring, which includes serum creatinine and potassium measurements, must have been reinforced in the present study population, as most patients were older than 60 and were susceptible to take concomitant medications and therefore, to require a spironolactone dose adjustment. For example, non-steroidal anti-inflammatory drugs (NSAIDs) prescribed in rheumatoid arthritis and in other immune diseases may exacerbate hyperkalemia when used with potassium sparing diuretics such as spironolactone [16,17]. However, these monitoring measurements are not available in RAMQ data. As the purpose of the present study was to assess the global incidence of gynecomastia and hyperkalemia, treatment regimen has not been considered. Further studies are needed to evaluate the impact of the treatment regimen on the incidence of these side effects in Canadian real-life setting. Furthermore, although digitalis, diuretics and nitrates are also used in severe HF, they have not been considered in the present study. However, as there is ample evidence supporting the use of β-blockers, ACE-I and/or ARB in patients with heart failure, which include current guidelines [18-20], results of this analysis could give a good estimate of spironolactone use in a real-life context despite study limitations.

## Conclusion

The analysis results suggest that the use of spironolactone is associated with an incidence of adverse events, which may have an impact on treatment compliance.

## Competing interests

At the time of the manuscript was prepared, Elodie Ramos was an employee of Pfizer Canada Inc. Jean Lachaine and Catherine Beauchemin had no competing interests.

## Authors' contributions

JL and CB designed the study, acquired, analyzed and interpreted the data, and drafted the article. ER revised the manuscript for important intellectual content. All authors read and approved the final manuscript.

## Pre-publication history

The pre-publication history for this paper can be accessed here:

http://www.biomedcentral.com/1472-6904/11/4/prepub
